# Hypocomplementemia is associated with worse renal survival in ANCA-positive granulomatosis with polyangiitis and microscopic polyangiitis

**DOI:** 10.1371/journal.pone.0195680

**Published:** 2018-04-05

**Authors:** Samuel Deshayes, Achille Aouba, Kathy Khoy, Delphine Mariotte, Thierry Lobbedez, Nicolas Martin Silva

**Affiliations:** 1 Department of Internal Medicine, CHU de Caen, Caen, France; 2 Department of Immunology, CHU de Caen, Caen, France; 3 Department of Nephrology, CHU de Caen, Caen, France; Keio University, JAPAN

## Abstract

Recent data suggest the existence of a complement alternative pathway activation in the pathogenesis of antineutrophilic cytoplasmic antibody (ANCA)-associated vasculitis (AAV), a condition that remains poorly understood. This study aims to assess the clinical characteristics and outcomes of granulomatosis with polyangiitis (GPA) and microscopic polyangiitis (MPA) patients with regard to their plasma complement levels at diagnosis. A retrospective monocentric study carried out at Caen University Hospital led to the identification of proteinase-3- or myeloperoxidase-ANCA-positive GPA and MPA patients from January 2000 to June 2016 and from September 2011 to June 2016, respectively. All patients with available C3 and C4 levels at diagnosis were included. Patients were categorized in the hypocomplementemia group if their C3 and/or C4 levels at diagnosis were below the lower limit of the normal range. Among the 76 AAV patients (43 GPA, 33 MPA), 4 (5%) had hypocomplementemia, and the 72 remaining patients exhibited normal plasma complement levels. All 4 hypocomplementemia patients had renal involvement. Hypocomplementemia was followed in 1 patient whose post-treatment complement level normalized within 1 month. Among all clinical and ANCA specificity, including relapse-free survival (p = 0.093), only overall and renal survival rates were significantly lower in the hypocomplementemia group (p = 0.0011 and p<0.001, respectively). Hypocomplementemia with low C3 and/or C4 levels at GPA or MPA diagnosis may be responsible for worse survival and renal prognosis. These results argue for larger and prospective studies to better determine the epidemiology of the disease and to assess complement-targeting therapy in these patients.

## Introduction

Among primitive systemic vasculitis associated with antineutrophilic cytoplasmic antibody (ANCA), granulomatosis with polyangiitis (GPA), microscopic polyangiitis (MPA) and eosinophilic granulomatosis with polyangiitis (EGPA) have been distinguished based on different clinical and pathophysiologic features that remain poorly understood [1]. There is, however, growing evidence through *in vitro*, murine models and immunopathological data supporting the involvement of complement alternative pathway activation in both GPA and MPA [2].

The first evidence for this finding came from *in vitro* studies. Proteinase-3 (PR3)-ANCA or myeloperoxidase (MPO)-ANCA-activated neutrophils can release complement alternative pathway activating factors [3,4]. C5a, resulting from complement activation via the cleavage of C5, allows neutrophil priming by engaging C5a receptors, leading to the attraction of additional neutrophils and the surface expression of ANCA antigens, thereby enabling neutrophils to be further activated by ANCA [4,5]. As a result, plasma and urinary levels of C5a are significantly higher in patients with active ANCA-associated vasculitis (AAV) compared to those in remission [6]. ANCA-induced neutrophil extracellular trap formation induces alternative complement pathway activation, which, at least in part, mediates endothelial cell damage [7].

Additional evidence comes from animal models of MPO-ANCA AAV. Mice with impaired complement alternative pathway are protected from anti-MPO-induced disease, including mice receiving anti-C5 or complement depletion treatment, or mice with genetic knock-out of C5, C5a receptor or Factor B, while knock-out mice for C4 or C6 are not protected [3,4,8,9]. Similarly, treatment with the human C5a receptor antagonist CCX168 (avacopan) leads to improvement in anti-MPO-induced lesions in mice carrying the corresponding knocked-in complement activated component [9].

Finally, mild deposits of immunoglobulins and/or complement components in glomeruli and small arteries on renal biopsies of AAV patients can be found in up to 54% of patients [10–12]. Additionally, systemic complement activation stigmas are observed with increased plasma and urine levels of C3a, C5a, and soluble C5b-9, increased plasma levels of Bb (but not C4d) and decreased plasma levels of properdin in AAV patients with active disease, in comparison to those in remission and healthy controls [13,14]. Moreover, the plasma level of factor Bb in patients with active AAV is correlated to the Birmingham Vasculitis Activity Score (BVAS) and the proportion of crescents at renal biopsy [13]. Bb deposits on renal biopsies are also correlated with renal lesions as measured by the extent of interstitial infiltrate, interstitial fibrosis and tubular atrophy [14]. Moreover, plasma complement factor H levels are significantly lower in active AAV; are inversely correlated with initial serum creatinine, BVAS, proportion of total crescents and cellular crescents in renal biopsies; and are independently associated with the composite outcome of end-stage renal disease (ESRD) or death [15]. The functional activities of factor H are also diminished in AAV patients through a decreased binding affinity for C3b; a decrease in decay-accelerating activity and cofactor activity for the factor I-mediated cleavage of C3b; and less efficient binding to monomeric C-reactive protein and endothelial cells [16]. In contrast, the expression levels of the membrane-bound complement regulatory proteins CD46, CD55, and CD59 are significantly lower in renal biopsy specimens of AAV patients compared to controls [17]. Taken together, the rationale for complement-targeted therapy shows promising results in GPA and MPA with positive ANCA. Eculizumab, the first engineered anti-C5a monoclonal antibody, has thus been tried with success as a rescue treatment in an AAV patient with clear activation of the complement alternative pathway [18]. Avacopan (CCX168), a C5a receptor (C5aR) inhibitor, also recently showed excellent and safe preliminary outcomes in replacing high-dose glucocorticoids in AAV patients in a randomized, placebo-controlled trial [19].

Nevertheless, few AAV patients among those tested show hypocomplementemia at diagnosis. However, recent studies have correlated low complement levels to unfavourable outcomes [10,20–24]. We therefore aimed to assess the correlation between the clinical characteristics and treatment outcomes as well as the levels of plasma complement at diagnosis of our AAV patient cohort.

## Materials and methods

### Study design

A single-centre retrospective monocentric study was performed at Caen University Hospital to identify an AAV cohort of patients with low plasma complement levels. All patients who tested positive for PR3-ANCA or MPO-ANCA from January 2000 to June 2016 and from September 2011 to June 2016, respectively, were included. The demographic, clinical, and treatment outcome data were collected from the patients’ clinical files.

The patients’ data were anonymized in databases before the authors were granted access. Given that the data were extracted from patients’ files, written informed consent was not required. In accordance with the French Public Health Law (Art. L 1121-1-1, Art. L 1121-1-2), formal approval from an Ethics Committee was not required for this type of study. The study was conducted in accordance with the recommendations of the Declaration of Helsinki and complied with the requirements of the French Commission Nationale de l’Informatique et des Libertés (DC-2008-559).

### Patient selection and disease dual classification according to clinical parameters and complement levels

All patients were diagnosed as having AAV based on the Chapel Hill Consensus Conference criteria [1]. AAV patients were classified as either GPA or MPA according to the European Medicines Agency vasculitis algorithm [25], and as the limited or severe form according to the Wegener’s Granulomatosis Etanercept Trial Research Group [26]. ESRD was defined as the onset of chronic dialysis or renal transplantation. PR3- and MPO-ANCA were determined by ELISA. C3 and C4 levels were assessed by nephelometry (Behring BNII nephelometer). Only GPA and MPA patients with available C3 and C4 plasma levels that were obtained before any immunosuppressive treatment were included. Patients were categorized in the hypocomplementemia group if the C3 and/or C4 levels at diagnosis were below the lower limit of the normal range, as supplied by the manufacturer (750–1,400 mg/l and 100–340 mg/l, respectively); otherwise, they were categorized in the normal complementemia group. Moreover, because both C3 and C4 are acute phase proteins and therefore are synthesized at increased levels during inflammation, which is common in AAV, their levels may be artificially normal, thereby masking a real increased complement activation in this setting. Therefore, we also divided AAV patients into two other groups according to the median level of serum complement levels observed in the whole cohort for C3 and C4, as previously reported [20]. The ANCA-associated vasculitis activity was determined using BVAS version 3 [27].

### Treatment classification and outcomes

Treatment was defined as follows: 1) “standard regimen” corresponding to the usual biphasic schedule with induction of remission with cyclophosphamide or rituximab associated with corticosteroids followed by maintenance immunosuppressive therapy 2) “moderate regimen” where patients did not receive a usual induction therapy, and was limited to methotrexate, azathioprine or mycophenolate mofetil, with or without association with corticosteroids and corticosteroids alone.

Relapse was defined as the reactivation of vasculitis in any organ system that required a change in therapy.

### Statistical methods

Categorical variables were reported as percentages and compared using the χ^2^ or Fisher’s tests, according to expected frequencies. Continuous variables were expressed as medians and interquartile ranges (IQR) and analysed using Student’s t-test. Patient data were censored at the time of relapse, death, or last follow-up visit, whichever occurred first. Associations between global survival, death-censored renal survival and relapse-free survival, and low plasma complement levels were analysed using Kaplan-Meier survival curves, and between-group differences were evaluated by the log-rank test. A p-value <0.05 was considered statistically significant.

## Results

Among the 157 AAV patients identified during the prespecified period, 81 were excluded (8 EGPA, 73 lacking C3 and C4 determinations before treatment initiation, Table 1). GPA and MPA excluded patients were younger (p = 0.03), had more frequently renal involvement (p = 0.04), were more likely to require dialysis (p = 0.003) and were followed longer (62 months, IQR 35–111 compared to 38 months, IQR 16–74.5, p = 0.02) than included patients. Renal survival was significantly lower in the excluded patients (p = 0.03), but overall and relapse-free survival were similar (p = 0.93 and 0.33, respectively, data not shown).

**Table 1:** Comparison of clinical characteristics and ANCA specificity between patients included or excluded, based on the availability of C3 and C4 plasma levels obtained before any immunosuppressive treatment (M/F: male/female; IQR: interquartile ranges).

	Patients included (n = 76)	Patients excluded (n = 73)	p value
Sex-ratio	1	1.5	0.21
Age at diagnosis (years), median (IQR)	65 (53–71.5)	60 (47–70)	0.03
BVAS, median (IQR)	18 (14–22)	18 (14–22)	0.75
Constitutional symptoms, n (%)	56 (73.7%)	54 (74%)	0.97
Pulmonary involvement, n (%)	53 (69.7%)	53 (72.6%)	0.7
Renal involvement, n (%)	50 (65.8%)	59 (80.8%)	0.04
Rheumatologic involvement, n (%)	43 (56.6%)	36 (49.3%)	0.38
Ear-nose-throat involvement, n (%)	38 (50%)	36 (49.3%)	0.94
Skin involvement, n (%)	14 (18.4%)	14 (19.2%)	0.91
Neurologic involvement, n (%)	12 (15.8%)	19 (26%)	0.13
Granulomatosis with polyangiitis, n (%)	45 (59.2%)	47 (64.4%)	0.52
PR3-ANCA, n (%)	45 (59.2%)	39 (53.4%)	0.48
Limited disease, n (%)	20 (26.3%)	13 (17.8%)	0.22
No usual induction treatment, n (%)	16 (21.1%)	13 (17.8%)	0.62
Plasma exchange, n (%)	18 (23.7%)	17 (23.3%)	0.96
End-stage renal disease, n (%)	14 (18.4%)	30 (41.1%)	0.003
Death, n (%)	12 (15.8%)	17 (23.3%)	0.25
Relapse, n (%)	27 (35.5%)	30 (41.1%)	0.49
Follow-up (months), median (IQR)	38 (16–74.5)	62 (35–111)	0.02

In total, 76 AAV patients were included, including 45 GPA and 31 MPA (Table 2). The median age at diagnosis was 65 years old (IQR, 53–71.5), with a sex ratio of 1. Clinical presentations included constitutional symptoms (56, 73.7%), pulmonary (53, 69.7%), renal (50, 65.8%), rheumatologic (43, 56.6%), and ear, nose or throat (38, 50%) involvement. The median BVAS was 18 (IQR, 14–22). After a median follow-up of 38 months (IQR, 16–74.5), 42 relapses in 27 patients (35.5%) and 12 deaths (15.8%) were noted.

**Table 2:** Clinical characteristics and ANCA specificity of granulomatosis with polyangiitis and microscopic polyangiitis patients, according to low or normal plasma complement levels (absolute values) performed before treatment (M/F: male/female; IQR: interquartile ranges).

	Hypocomplementemia group (n = 4)	Normal complement level group (n = 72)	p value
Sex-ratio	1	1	1
Age at diagnosis (years), median (IQR)	71.5 (66–78.75)	65 (52.5–71)	0.18
BVAS, median (IQR)	20.5 (18.5–23.5)	18 (13.75–22)	0.38
Constitutional symptoms, n (%)	3 (75%)	53 (73.6%)	1
Pulmonary involvement, n (%)	4 (100%)	49 (68.1%)	0.31
Renal involvement, n (%)	4 (100%)	46 (63.9%)	0.3
Rheumatologic involvement, n (%)	2 (50%)	41 (56.9%)	1
Ear-nose-throat involvement, n (%)	2 (50%)	36 (50%)	1
Skin involvement, n (%)	0	26 (36.1%)	0.3
Neurologic involvement, n (%)	1 (25%)	21 (29.2%)	1
Granulomatosis with polyangiitis, n (%)	3 (75%)	42 (58.3%)	0.65
PR3-ANCA, n (%)	2 (50%)	43 (59.7%)	1
Limited disease, n (%)	0	20 (27.8%)	0.57
No usual induction treatment, n (%)	0	16 (22.2%)	0.58
Plasma exchange, n (%)	3 (75%)	15 (20.8%)	0.04
End-stage renal disease, n (%)	3 (75%)	11 (15.3%)	0.019
Death, n (%)	2 (50%)	10 (13.9%)	0.12
Relapse, n (%)	1 (25%)	26 (36.1%)	1
Follow-up (months), median (IQR)	9 (1–17)	42.5 (16–76.25)	0.07

**Fig 1:**
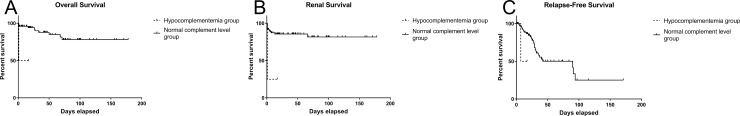
**Kaplan-Meier estimate of overall (A), renal (B) and relapse-free (C) survival rates in patients with granulomatosis with polyangiitis or microscopic polyangiitis, according to low or normal plasma complement levels (absolute values) performed before treatment (p = 0.0011, <0.001 and 0.093, respectively)**.

In total, 4 of the 76 patients (5.3%) had hypocomplementemia: 1 patient had isolated low C3 level, 1 had isolated low C4 level, and 2 had both. All 4 patients had renal involvement with more frequent ESRD in comparison to the remaining 72 patients (p = 0.019), and had to be treated more frequently with plasma exchange treatment (p = 0.04). At AAV diagnosis these patients had hematuria (n = 3) and proteinuria (n = 3, between 1.6 and 3.2 g/d), with elevated creatinine levels (234, 600, 654 and 1080 μmol/l, corresponding to creatinine clearance of 16, 7.1, 5.2 and 3.8 ml/min/1.73m^2^ according to the Chronic Kidney Disease Epidemiology Collaboration (CKD-EPI) equation, respectively). Both overall and renal survival were significantly lower in the hypocomplementemia group of four patients (p = 0.0011 and p<0.001, respectively, Fig 1), but relapse-free survival was similar (p = 0.093) in both groups. There were no other significant differences between the groups (Table 2) for various types of organ involvement, ANCA type, disease clinical classification as severe or limited or the type of treatment, which is dependent on severe versus limited status. The initial low level of complement, controlled in 1 patient, became normal 1 month after treatment was initiated. No clinical thrombotic microangiopathy or infectious features were found in the 4 patients with initial hypocomplementemia. Two patients had a kidney biopsy, and both revealed crescentic glomerulonephritis, with tubular necrosis and interstitial fibrosis (50% of the cortical interstitium) in one patient. On the contrary, no histologic features of thrombotic microangiopathy were observed on the two kidney biopsies.

**Fig 2:**
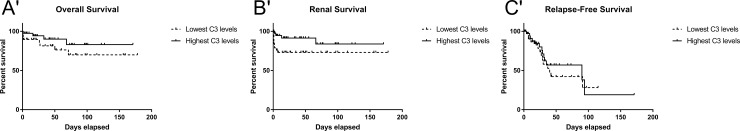
**Kaplan-Meier estimate of overall (A'), renal (B') and relapse-free (C') survival rates in patients with granulomatosis with polyangiitis or microscopic polyangiitis, according to the low normal or high normal plasma C3 levels (relative values) performed before treatment, based on the median value of C3 levels observed in the whole cohort (p = 0.23, 0.07 and 0.60, respectively)**.

There were no statistically significant differences between the clinical characteristics, ANCA specificity and treatment when comparing the groups based on median C3 level, except for a lower rate of intensive regimen treatment in the group with the low normal C3 level (Table 3). Renal, relapse-free and overall survival rates were not significantly different between the 2 groups (p = 0.07, p = 0.60 and p = 0.23, respectively, Fig 2).

**Table 3:** Clinical characteristics and ANCA specificity of granulomatosis with polyangiitis and microscopic polyangiitis patients, according to the low normal or high normal plasma C3 levels (relative values) performed before treatment, based on the median value of C3 levels observed in the whole cohort (M/F: male/female; IQR: interquartile ranges).

	Low normal C3 levels (n = 38)	High normal C3 levels (n = 38)	p value
Sex-ratio (M/F)	1.24	0.81	1
Age at diagnosis (years), median (IQR)	66.5 (54.25–74.75)	65 (51–70.5)	0.48
BVAS, median (IQR)	17 (13.25–21)	18.5 (14–23)	0.28
Constitutional symptoms, n (%)	25 (65.8%)	31 (81.6%)	0.12
Pulmonary involvement, n (%)	23 (60.5%)	30 (78.9%)	0.09
Renal involvement, n (%)	26 (68.4%)	24 (63.2%)	0.63
Rheumatologic involvement, n (%)	19 (50%)	24 (63.2%)	0.25
Ear-nose-throat involvement, n (%)	15 (39.5%)	23 (60.5%)	0.07
Skin involvement, n (%)	12 (31.6%)	14 (36.8%)	0.63
Neurologic involvement, n (%)	10 (26.3%)	12 (31.6%)	0.62
Granulomatosis with polyangiitis, n (%)	19 (50%)	26 (68.4%)	0.11
PR3-ANCA, n (%)	19 (50%)	26 (68.4%)	0.11
Limited disease, n (%)	12 (31.6%)	8 (21.1%)	0.30
No usual induction treatment, n (%)	12 (31.6%)	4 (10.5%)	0.03
Plasma exchange, n (%)	8 (21.1%)	10 (26.3%)	0.59
End-stage renal disease, n (%)	10 (26.3%)	4 (10.1%)	0.08
Death, n (%)	8 (21.1%)	4 (10.1%)	0.21
Relapse, n (%)	14 (36.8%)	13 (34.2%)	0.82
Follow-up (months), median (IQR)	39 (9.5–74)	38 (16.25–75.25)	0.98

**Fig 3:**
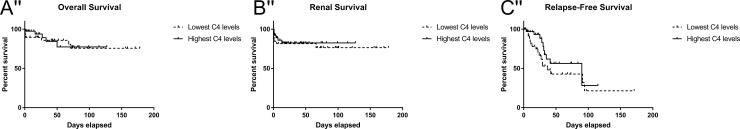
**Kaplan-Meier estimate of overall (A''), renal (B'') and relapse-free (C'') survival rates in patients with granulomatosis with polyangiitis or microscopic polyangiitis, according to the low normal or high normal plasma C4 levels (relative values) performed before treatment, based on the median value of C4 levels observed in the whole cohort (p = 0.83, 0.69 and 0.20, respectively)**.

When comparing the groups according to median C4 level, the low normal C4 group was associated with a higher BVAS score (p = 0.04). There were no other statistically significant differences between the groups with regard to the other study parameters (Table 4). Similar to the median C3 level classification, renal, relapse-free and overall survival rates were not significantly different between the low normal and high normal C4 groups based on median C4 level (p = 0.69, p = 0.20 and p = 0.83, respectively, Fig 3).

**Table 4:** Clinical characteristics and ANCA specificity of granulomatosis with polyangiitis and microscopic polyangiitis patients, according to the low normal or high normal plasma C4 levels (relative values) performed before treatment, based on the median value of C4 levels observed in the whole cohort (M/F: male/female; IQR: interquartile ranges).

	Low normal C4 levels (n = 38)	High normal C4 levels (n = 38)	p value
Sex-ratio (M/F)	1.1	0.9	0.65
Age at diagnosis (years), median (IQR)	67.5 (56.25–77)	62 (50.75–69.75)	0.07
BVAS, median (IQR)	21 (14–24)	16.5 (12.25–21)	0.04
Constitutional symptoms, n (%)	31 (81.6%)	25 (65.8%)	0.12
Pulmonary involvement, n (%)	29 (76.3%)	24 (63.2%)	0.22
Renal involvement, n (%)	28 (73.7%)	22 (57.9%)	0.15
Rheumatologic involvement, n (%)	21 (55.3%)	22 (57.9%)	0.82
Ear-nose-throat involvement, n (%)	18 (47.4%)	20 (52.6%)	0.65
Skin involvement, n (%)	15 (39.5%)	11 (28.9%)	0.34
Neurologic involvement, n (%)	11 (28.9%)	11 (28.9%)	1
Granulomatosis with polyangiitis, n (%)	25 (65.8%)	20 (52.6%)	0.25
PR3-ANCA, n (%)	25 (65.8%)	20 (52.6%)	0.25
Limited disease, n (%)	9 (23.7%)	11 (28.9%)	0.61
No usual induction treatment, n (%)	5 (13.2%)	11 (28.9%)	0.10
Plasma exchange, n (%)	10 (26.3%)	8 (21.1%)	0.59
End-stage renal disease, n (%)	8 (21.1%)	6 (15.8%)	0.56
Death, n (%)	7 (18.4%)	5 (13.2%)	0.53
Relapse, n (%)	17 (44.7%)	10 (26.3%)	0.10
Follow-up (months), median (IQR)	49 (17.5–93)	30 (10.75–50)	0.04

## Discussion

This study found, first, a low proportion of approximately 5% of patients with hypocomplementemia at diagnosis among 76 GPA and MPA patients, and second, that these patients had a worse overall and death-censored renal survival rates. Moreover, these features corresponded to patients who exhibited hypocomplementemia as determined by the absolute values of C3 and/or C4 falling below the lower limit of the normal range, but not when patients were categorized based on the median value of complement levels. Apart from the worse renal survival in patients with low plasma C3 and/or C4 levels, no other clinical or biological differences were found that could explain the worse overall survival. Although the low normal C4 level group exhibited a higher BVAS score than that of the high normal C4 level group, no significant clinical or survival differences were found between the two groups.

Worse renal survival may be related to higher rates of thrombotic microangiopathy, immune complex deposits and/or chronic lesions in renal biopsies in AAV patients with hypocomplementemia [10,23]. Our limited number of patients with hypocomplementemia and of those who underwent renal biopsy in this setting does not allow us to assess these hypotheses, but that of thrombotic microangiopathy appears unlikely because it is typically easy to recognize.

Because of the retrospective design of this study, less than half of our patients had C3 and C4 level determination at diagnosis, which could have resulted in a selection bias. Indeed, as shown in Table 1, the excluded population was not similar to the included patients, mainly with more frequent renal involvement, more patients requiring dialysis and a worse renal survival. The median follow-up period was short (38 months). Another limitation is related to the method of patient selection that excluded those with ANCA-negative AAV. Therefore, patients with limited disease, which may be more frequently ANCA-negative, may be under-represented in this study [28]. However, as hypocomplementemia was associated with worse overall and death-censored renal survival rates in our study, the inclusion of ANCA-negative patients, assuming they exhibit the limited pattern of the diseases, would not change these two findings. Still, this assumption should be analysed in further studies. However, a different proportion of patients with hypocomplementemia could be found using a different typology of included patients. Indeed, a previous Japanese study of 81 AAV patients that also included 11 EGPA and 3 renal-limited vasculitis patients found a higher proportion of 20% of patients with hypocomplementemia (using absolute values) and no association between the hypocomplementemia status and worse renal prognosis [10]. Moreover, unlike our study, hypocomplementemia was associated with a higher rate of skin lesions, diffuse alveolar haemorrhage, thrombotic microangiopathy, and immune complex deposits in renal biopsies. Also in this Japanese study, complement level remained low under treatment in 7 out of 11 patients who exhibited hypocomplementemia at diagnosis. Nevertheless, this study found similar results to ours in that hypocomplementemia at AAV diagnosis was not associated with worse relapse-free survival but with worse overall survival [10].

In another French retrospective study on 45 cases of AAV, no patient exhibited hypocomplementemia. Therefore, patients were divided into two groups according to the median C3 level. Patients in the low normal C3 level group also had lower C4 levels, lower eGFR at diagnosis, and poorer long-term and death-censored renal survival, but no difference in relapse-free survival. Conversely, such associations were not found when patients were analysed according to serum C4 levels [20].

An Israeli study of 30 AAV patients that also included 7 EGPA patients found 20% and 3.3% of patients with low C3 and low C4 levels, respectively, which normalized under treatment [21]. A low C3 level, unlike in our study, was associated with older age, anti-MPO antibodies, a higher rate of GPA, antinuclear antibody positivity, higher C-reactive protein levels, lower haemoglobin levels, lower estimated glomerular filtration rate (eGFR) at presentation, and lower eGFR at last visit. A low C3 level was also associated with decreased renal function at the last study visit (defined as eGFR <60 ml/min/1.73 m^2^) and patient survival, similar to our results [21]. In an Italian study of 46 AAV patients with renal involvement, a low C3 level was found in 14/40 (35%) patients, and a low C4 level was found in 1 patient (2%) [22]. If patients with a low C3 level were older, unlike our study, they similarly exhibited a worse death-censored renal survival.

A study by Villacorta *et al*. on 111 AAV Spanish patients (including 58 with renal limited vasculitis) found a low proportion of 8.1% of patients with a low C3 level at diagnosis, which is close to that of our study. Patients were divided into three tertiles according to C3 concentration [23]. The group with the lowest C3 levels was associated with a higher need for dialysis and the lowest response rate to treatment as well as the highest presence of chronic lesions at the initial biopsy. The lowest C3 level appeared to be an independent risk factor for ESRD and death. Conversely, no difference was found in renal and overall survival rates between patients with respect to C4 level [23]. Finally, a recent Croatian study by Crnogorac *et al*. also found in 77 consecutive patients with pauci-immune crescentic glomerulonephritis (including 13 with renal limited vasculitis) a low percentage (12%) of patients with a low C3 level at diagnosis, which was associated with lower overall and renal survival rates (hazard ratio: 4.315 [1.350–3.799] and 3.679 [1.144–11.827] on multivariate analysis, respectively), similar to our study [24].

In all of these studies, including ours, the C4 level does not appear to influence overall or organ outcomes, contrary to what was found with the C3 level. However, very few AAV patients exhibit a low C4 level at diagnosis. In addition to the alternative pathway, these findings would support the involvement of another complement pathway in the pathogeny of some AAV cases, possibly including a pathological activation of the classical pathway. Indeed, the urinary level of C1q has been found to be significantly higher in some patients with active AAV compared to controls [14].

To conclude, hypocomplementemia related to low C3 and C4 levels at diagnosis appears to be associated with worse overall and death-censored renal survival rates in ANCA-positive GPA and MPA patients, but it is not associated with the intensity of disease activity as it is currently assessed. This particular phenotype may confer a poorer effectiveness to usual immunosuppressive approaches. Corresponding patients may greatly benefit from complement-targeting therapy, as in other complement-mediated diseases such as thrombotic microangiopathies that also frequently exhibit renal involvement. These results therefore argue for larger and prospective studies.
